# Effects of Access Cavity Design on the Shaping Ability and Dentine Thickness Following Canal Preparation Using XP‐Endo Shaper or Reciproc

**DOI:** 10.1111/aej.70045

**Published:** 2025-12-05

**Authors:** Renata Muniz Alvez Cruz, Ana Flávia Almeida Barbosa, Carolina Oliveira de Lima, Ricardo Tadeu Lopes, Marco Aurélio Versiani, Emmanuel João Nogueira Leal da Silva, Luciana Moura Sassone

**Affiliations:** ^1^ Departament of Endodontics State University of Rio de Janeiro Rio de Janeiro Brazil; ^2^ Department of Dentistry Federal University of Juiz de Fora Governador Valadares Brazil; ^3^ Nuclear Engineering Program, Federal University of Rio de Janeiro (UFRJ) Rio de Janeiro Brazil; ^4^ Oral Health Center Brazilian Military Police Minas Gerais Brazil; ^5^ Department of Endodontics, School of Dentistry Grande Rio University (UNIGRANRIO) Rio de Janeiro Brazil

**Keywords:** access cavity, mandibular molar, micro‐CT, minimally invasive, nickel‐titanium instruments, root canal preparation

## Abstract

This study compared the shaping ability and remaining dentine thickness in mandibular molars prepared with traditional (TradAC) or ultraconservative (UltraAC) access cavities using Reciproc or XP‐endo Shaper instruments. Thirty‐two molars were scanned with micro‐CT before and after preparation to assess canal surface, volume, untouched walls, and dentine thickness. Both access cavity designs showed a significant reduction in dentine thickness after preparation, but no differences were found between TradAC and UltraAC in either mesial or distal roots (*p* > 0.05). In the distal root, Reciproc produced a greater reduction in dentine thickness compared with XP‐endo Shaper (*p* < 0.05), whereas shaping ability parameters did not differ significantly between instruments (*p* > 0.05). Some canals exhibited residual dentine thickness below 0.5 mm, particularly when preoperative thickness was already minimal. Within the limitations of this laboratory study, access cavity design had no influence on dentine preservation, while the choice of instrument affected dentine reduction only in the distal root.

## 1 Introduction

The traditional access cavity (TradAC) preparation in endodontics involves meticulously removing any decayed portions of the tooth, existing dental restorations, and the entire roof of the pulp chamber to create a straight line pathway to the root canal orifices [1]. A few years back, however, some authors hypothesized that the total removal of the pulp chamber roof (referred to as the ‘soffit’), coupled with excessive reduction of pericervical dentine—an area approximately 4 mm above and below the bone crest—might elevate the likelihood of cusp deflection and post‐endodontic treatment fracture in teeth [2]. Drawing from these insights, some clinicians have put forth a proposed solution to this issue in a non‐evidence‐based narrative paper, advocating for the application of minimally invasive principles to endodontic access preparation [3]. This approach seeks to address the challenges identified by adopting techniques and strategies that prioritise preserving natural tooth structure and minimising unnecessary removal, thereby potentially improving treatment outcomes and reducing the risk of complications. More specifically, the authors of this study suggested a preservation approach focused on retaining the majority of the soffit and pericervical dentine. By prioritising the conservation of these components, they sought to keep the fracture resistance of the tooth, potentially reducing the risk of fractures and enhancing overall treatment outcomes [3]. In subsequent years, the adoption of minimal access cavity techniques marked a growing trend in endodontic practice and various alternative cavity designs were suggested without validation or empirical support [4].

The ultraconservative access cavity (UltraAC) approach aims to preserve as much of the pulp chamber roof as possible by minimising the size of the access preparation [5]. This conservative strategy was proposed to enhance the structural integrity of the tooth, ultimately reducing the risk of fracture in endodontically treated teeth [6, 7]. However, more robust evidence is still needed to confirm the role of minimally invasive access in treatment outcomes. Laboratory research suggests that adopting the UltraAC approach may potentially compromise important aspects of endodontic procedures, such as thorough cleaning, effective disinfection, and accurate canal orifice location, primarily due to coronal interference [8, 9, 10]. Moreover, UltraAC may inadvertently subject endodontic instruments to heightened pressure, especially in highly curved canals [11, 12, 13, 14]. This increased force can result in instrument fracture and significant wear on the inner canal walls [11, 12, 15, 16]. This latter concern is especially significant in treating mandibular molars, as they contain a vulnerable region referred to as the ‘danger zone’, a thinner area of dentine, primarily located at the distal aspect of the mesial root [16, 17]. Excessive removal of dentine and unintentional alteration of canal shape, often resulting from the use of large‐tapered and less flexible instruments, can alter the natural shape of the canal, and may adversely impact the ability of the tooth to withstand fracture [18, 19]. Therefore, instruments with reduced taper should be considered to reduce the removal of dentinal tissue. Although the optimal dentine thickness required to ensure maximal fracture resistance remains uncertain [20], preserving the original geometry of root canals is considered essential for ensuring the structural integrity of the tooth [19]. Among these systems, the XP‐endo Shaper (FKG, La Chaux‐de‐Fonds, Switzerland) stands out for its unique MaxWire alloy, which responds to body temperature (~35°C) by expanding from an initial taper of 0.01 to at least 0.04. According to the manufacturer, this temperature‐induced transformation enables the instrument to adapt dynamically to the canal morphology, aligning with the principles of minimally invasive endodontics.

In teeth with significant morphological complexities, such as mandibular molars, the potential advantages of minimally invasive endodontic techniques in maintaining structural integrity are especially compelling. However, despite the clinical importance of such approaches, there exists a lack of comprehensive data regarding their actual effectiveness and practical benefits. This study aimed to compare the shaping ability and remaining dentine thickness in the pericervical area of mandibular molars prepared with either a TradAC or an UltraAC, using the conventional Reciproc instrument and the XP‐endo Shaper—an instrument specifically designed to preserve canal structure—providing a comprehensive evaluation of how each instrument adapts to different access designs while maintaining canal integrity. The null hypothesis tested proposed that there would be no difference in remaining dentine thickness or preparation protocols based on the type of access cavity.

## 2 Material and Methods

The manuscript of this laboratory study has been written according to Preferred Reporting Items for Laboratory Studies in Endodontology (PRILE) 2021 guidelines (Figure S1).

### 2.1 Sample Size Calculation

The sample size was estimated based on a previous study [21], with statistical parameters including a power of 95%, a significance level of 5%, and an effect size of 3.74, which were input into a family of *F*‐tests for one‐way ANOVA analysis (G*Power software v.3.1.7; Heinrich Heine, Universität Düsseldorf). After analysis, a total sample size of 32 was considered optimal, with 8 samples allocated to each group, to ensure adequate statistical power to detect meaningful differences between the groups.

### 2.2 Sample Selection and Imaging

After approval of the research protocol by the local Ethics Committee (48541321.8.0000.5259), 82 rooted mandibular molars, extracted within 6 months of the experimental procedures for reasons unrelated to this study, were cleaned and subsequently stored in distilled water. They exhibited fully formed roots of similar length and a moderate degree of curvature (5°–20°), and showed no signs of fracture, resorption, or calcifications. Each tooth underwent scanning using a micro‐CT device (SkyScan 1173; Bruker‐microCT, Kontich, Belgium) configured with settings at 70 kV, 114 mA, and a pixel size of 20 μm. The exposure time was 272 ms, with frame averaging of 4, and a 360° rotation around the vertical axis in increments of 0.5°, employing a 1‐mm thick aluminium filter. Following reconstruction using standardised parameters (NRecon v.1.6.1.0; Bruker‐microCT), the obtained datasets were assessed to evaluate the root canal configuration and to calculate the surface area (mm^2^) and volume (mm^3^) of the root canals, as well as the minimal dentine thickness towards the distal and mesial directions relative to each canal at the furcation area using CTan v1.6.6.0 (Bruker‐microCT). From the initial sample, 32 teeth exhibiting a single distal canal and a mesial canal with an isthmus Type V (complete communication between the two canals) [22] were matched based on previously calculated parameters. These teeth were then distributed into 2 groups (*n* = 16) according to the type of access (TradAC or UltraAC). Each group was further subdivided into 2 groups (*n* = 8) based on the root canal preparation system using Reciproc (VDW, Munich, Germany) or XP‐endo Shaper (FKG, La Chaux‐de‐Fonds, Switzerland). TradAC and UltraAC access cavities were defined and prepared in accordance with previously published protocols [4, 5].

### 2.3 Preparation Protocols

Following the preparation of access cavities, a size 10 K‐file (Dentsply Sirona Endodontics, Ballaigues, Switzerland) was used to confirm apical patency and establish the working length (WL), set 1.0 mm short of the apical foramen. Subsequently, each tooth was mounted on a dental mannequin to simulate an ergonomic working position under conditions resembling clinical practice, and each root canal was irrigated with 2 mL of 2.5% sodium hypochlorite (NaOCl). Following that, root canals were progressively enlarged using Reciproc or XP‐endo Shaper instruments. Reciproc was activated in the ‘RECIPROC ALL’ preset program of the VDW Silver motor (VDW). The mesial canals were enlarged using Reciproc R25 (25/0.08v), while the distal canal was initially enlarged with Reciproc R25, followed by the Reciproc R40 (40/0.06v). Each instrument was employed up to the WL in a slow in‐and‐out pecking motion with a 3 mm amplitude. Following three pecking movements, the instrument was withdrawn from the canal and cleaned with alcohol. The XP‐endo Shaper instrument (30/0.01v) was activated at 800 rpm and 1 N·cm in rotary motion (VDW Silver motor; VDW). Five slow and smooth in‐and‐out motions were executed until the instrument reached the WL. After that, ten additional in‐and‐out motions were performed up to the WL in each root canal. A single instrument was used for the preparation of all canals within a single tooth and then discarded. In all groups, root canals were irrigated with a total of 10 mL of 2.5% NaOCl throughout the preparation procedures using a 30‐gauge Endo‐Eze needle (Ultradent Products Inc., South Jordan, UT, USA) positioned 2 mm short of the WL. Final irrigation was accomplished using 3 mL of 17% EDTA (1 min), followed by 3 mL of 2.5% NaOCl and a final flush with 2 mL of distilled water. All preparation procedures were carried out by a single experienced endodontist (R.M.A.C.) under magnification (×12.5; OPMI pico; ZEISS, Jena, Germany). After that, root canals were gently dried with paper points, and the teeth were rescanned using the same pre‐operative parameters.

### 2.4 Micro‐CT Analyses

The postoperative datasets of the teeth were coregistered with their respective preoperative datasets using 3D Slicer v. 5.6.1 software (www.slicer.org). Then, CTAn v.1.20.8 software (Bruker‐microCT) was used to evaluate the root canal surface area and volume, as well as the percentage of untouched canal walls in the mesial and distal roots. The percentage of untouched canal walls was calculated by the number of static voxels (voxels present in the same position on the canal surface before and after instrumentation) divided by the total number of voxels present on the root canal surface [23]. Shaping ability parameters were assessed in the apical third, specifically from the apical foramen up to 4 mm in the coronal direction, as well as from the foramen to 10 mm coronally, to ensure consistency across roots of varying lengths. To evaluate dentine thickness, it involved generating a 3D map, which was saved for structural thickness analysis. Colour‐coded cross‐sections were used to identify the direction and measure the smallest dentine thickness related to each root canal at 1.0‐mm intervals from 1 mm below the furcation level (level 1) up to 5 mm towards the apical direction (levels 2 to 5). Measurements (in mm) were taken from the boundary of each root canal towards the external root surface using the “line” tool in the CTAn v.1.20.8 software in the thinnest area of dentine thickness. This measurement process was conducted both before and after root canal preparation and was repeated three times at each level. The average of these results was considered the minimal dentine thickness for that specific level. Subsequently, the percentage reduction of dentine thickness was calculated for each root canal using the formula (DT_B_ − DT_A_/DT_B_)*100, where DT_B_ and DT_A_ are the dentine thickness before and after preparation. Qualitative comparisons of root thicknesses were conducted using 3D colour‐coded models of the matched roots in CTVox v.3.3.1 software (Bruker‐microCT).

### 2.5 Statistical Analysis

The data obtained for root canal area, volume, percentage of untouched canal walls, and dentine thickness in both mesial and distal canals was subjected to normality assessment using the Shapiro–Wilk test, with *p*‐values exceeding 0.05 indicating normal distribution. Group differences were evaluated based on their distribution using either one‐way ANOVA followed by Tukey's *post hoc* test or Kruskal‐Wallis followed by Student–Newman–Keuls *post hoc* test (BioStat v. 5.0.1; AnalystSoft). The results were presented as mean (standard deviation) and median [interquartile range]. Significance level was set at 5%.

## 3 Results

**FIGURE 1:**
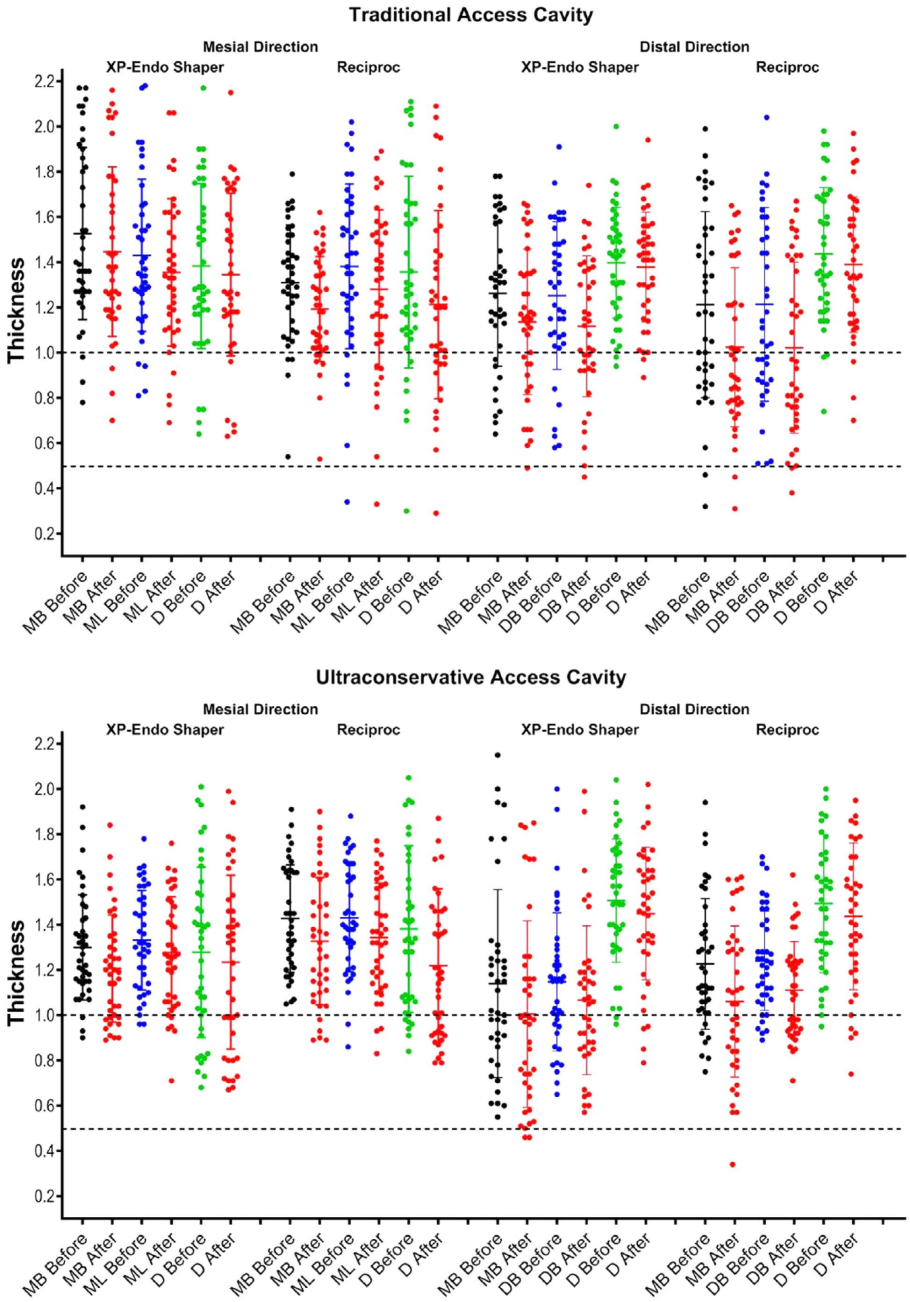
Graphics depicting the measurements of dentine wall thickness, in millimetres, in the mesial and distal directions before and after preparation of the mesiobuccal (MB), mesiolingual (ML), and distal (D) canals of 32 mandibular molars prepared using either TradAC (above) or UltraAC (below) using two preparation protocols.

**FIGURE 2:**
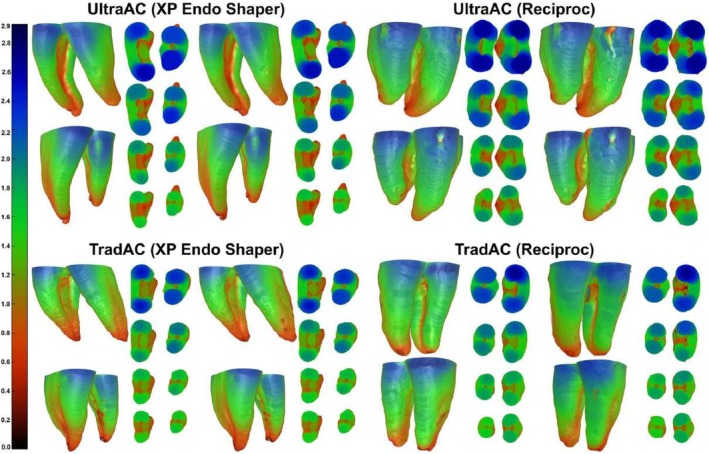
Representative three‐dimensional colour‐coded models displaying the dentine thickness on the mesial and distal aspects of the roots, before and after canal shaping with XP‐endo Shaper and Reciproc instruments in mandibular molars prepared with traditional or ultraconservative access cavities. Thick structures are indicated in blue and green, whilst areas of red indicated areas of thin dentine.

Measurements for the mesiobuccal, mesiolingual, and distal canals, under both access cavity protocols, are presented in Figure 1 and detailed in Tables 1 and 2. Statistical analyses confirmed the homogeneity of groups regarding the evaluated parameter at baseline (*p* > 0.05) and revealed a significant reduction in dentine thickness after root canal preparation in all levels within both groups (*p* < 0.05) (Figure 2; Tables 1 and 2).

**TABLE 1:** Mean (standard deviation) of dentine thickness (in mm) measured on the mesial aspect of the roots, before and after preparation of the mesiobuccal (MB), mesiolingual (ML) and distal (D) canals with XP‐endo Shaper and Reciproc instruments in mandibular molars prepared with traditional (TradAC) or ultraconservative (UltraAC) access cavities.

Level	Canal	TradAC (*n* = 16)				UltraAC (*n* = 16)			
		XP‐endo Shaper		Reciproc		XP‐endo Shaper		Reciproc	
		Before	After	Before	After	Before	After	Before	After
1	MB	1.70 ± 0.32	1.63 ± 0.33	1.50 ± 0.15	1.40 ± 0.12	1.60 ± 0.22	1.50 ± 0.22	1.65 ± 0.14	1.54 ± 0.18
	ML	1.62 ± 0.29	1.57 ± 0.28	1.56 ± 0.21	1.49 ± 0.23	1.55 ± 0.12	1.51 ± 0.12	1.64 ± 0.16	1.58 ± 0.15
	D	1.69 ± 0.37	1.63 ± 0.36	1.70 ± 0.34	1.50 ± 0.37	1.60 ± 0.31	1.52 ± 0.35	1.83 ± 0.36	1.60 ± 0.37
2	MB	1.49 ± 0.36	1.42 ± 0.33	1.32 ± 0.16	1.22 ± 0.17	1.35 ± 0.16	1.28 ± 0.17	1.48 ± 0.19	1.41 ± 0.23
	ML	1.39 ± 0.26	1.35 ± 0.25	1.40 ± 0.24	1.33 ± 0.26	1.35 ± 0.14	1.32 ± 0.15	1.51 ± 0.20	1.45 ± 0.17
	D	1.41 ± 0.34	1.37 ± 0.35	1.33 ± 0.30	0.96 ± 0.44	1.39 ± 0.34	1.34 ± 0.39	1.45 ± 0.32	1.23 ± 0.32
3	MB	1.52 ± 0.41	1.43 ± 0.40	1.24 ± 0.16	1.13 ± 0.13	1.23 ± 0.16	1.15 ± 0.14	1.40 ± 0.24	1.31 ± 0.28
	ML	1.42 ± 0.38	1.35 ± 0.36	1.40 ± 0.32	1.30 ± 0.33	1.34 ± 0.18	1.25 ± 0.28	1.42 ± 0.19	1.32 ± 0.19
	D	1.32 ± 0.35	1.29 ± 0.36	1.30 ± 0.37	1.17 ± 0.39	1.24 ± 0.36	1.21 ± 0.38	1.26 ± 0.26	1.10 ± 0.25
4	MB	1.51 ± 0.42	1.40 ± 0.38	1.26 ± 0.27	1.11 ± 0.24	1.16 ± 0.12	1.05 ± 0.12	1.31 ± 0.22	1.20 ± 0.29
	ML	1.39 ± 0.36	1.28 ± 0.34	1.30 ± 0.44	1.16 ± 0.40	1.25 ± 0.17	1.19 ± 0.19	1.31 ± 0.23	1.20 ± 0.22
	D	1.29 ± 0.35	1.26 ± 0.35	1.28 ± 0.43	1.17 ± 0.45	1.14 ± 0.40	1.12 ± 0.40	1.17 ± 0.25	1.06 ± 0.25
5	MB	1.42 ± 0.43	1.34 ± 0.45	1.23 ± 0.39	1.10 ± 0.33	1.16 ± 0.19	1.05 ± 0.19	1.29 ± 0.24	1.17 ± 0.31
	ML	1.33 ± 0.38	1.23 ± 0.36	1.25 ± 0.53	1.12 ± 0.45	1.16 ± 0.28	1.11 ± 0.28	1.27 ± 0.24	1.17 ± 0.24
	D	1.20 ± 0.28	1.18 ± 0.29	1.17 ± 0.52	1.10 ± 0.54	1.02 ± 0.23	0.99 ± 0.23	1.18 ± 0.20	1.10 ± 0.22

**TABLE 2:** Mean (standard deviation) of dentine thickness (in mm) measured on the distal aspect of the roots, before and after preparation of the mesiobuccal (MB), mesiolingual (ML) and distal (D) canals with XP‐endo Shaper and Reciproc instruments in mandibular molars prepared with traditional (TradAC) or ultraconservative (UltraAC) access cavities.

Level	Canal	TradAC (*n* = 16)				UltraAC (*n* = 16)			
		XP‐endo Shaper		Reciproc		XP‐endo Shaper		Reciproc	
		Before	After	Before	After	Before	After	Before	After
1	MB	1.34 ± 0.38	1.14 ± 0.37	1.46 ± 0.32	1.17 ± 0.27	1.35 ± 0.39	1.14 ± 0.36	1.47 ± 0.30	1.18 ± 0.40
	ML	1.38 ± 0.36	1.16 ± 0.34	1.53 ± 0.44	1.19 ± 0.39	1.31 ± 0.19	1.20 ± 0.21	1.54 ± 0.13	1.34 ± 0.16
	D	1.58 ± 0.15	1.57 ± 0.15	1.63 ± 0.18	1.59 ± 0.18	1.72 ± 0.21	1.69 ± 0.23	1.78 ± 0.30	1.74 ± 0.28
2	MB	1.26 ± 0.34	1.08 ± 0.34	1.26 ± 0.40	1.03 ± 0.36	1.14 ± 0.36	0.98 ± 0.35	1.17 ± 0.23	0.92 ± 0.37
	ML	1.26 ± 0.38	1.10 ± 0.38	1.22 ± 0.40	0.93 ± 0.38	1.16 ± 0.22	1.05 ± 0.28	1.25 ± 0.14	1.07 ± 0.22
	D	1.46 ± 0.18	1.44 ± 0.17	1.47 ± 0.19	1.45 ± 0.18	1.56 ± 0.23	1.53 ± 0.24	1.59 ± 0.28	1.58 ± 0.28
3	MB	1.27 ± 0.36	1.14 ± 0.35	1.18 ± 0.41	1.01 ± 0.36	1.02 ± 0.38	0.90 ± 0.41	1.09 ± 0.19	0.96 ± 0.26
	ML	1.26 ± 0.34	1.12 ± 0.32	1.16 ± 0.44	0.99 ± 0.41	1.08 ± 0.27	1.01 ± 0.31	1.12 ± 0.10	0.98 ± 0.18
	D	1.39 ± 0.20	1.38 ± 0.20	1.42 ± 0.21	1.37 ± 0.22	1.47 ± 0.22	1.41 ± 0.24	1.50 ± 0.27	1.46 ± 0.27
4	MB	1.22 ± 0.31	1.17 ± 0.32	1.15 ± 0.47	1.00 ± 0.39	1.14 ± 0.51	1.05 ± 0.53	1.20 ± 0.32	1.13 ± 0.33
	ML	1.19 ± 0.30	1.13 ± 0.29	1.12 ± 0.43	1.02 ± 0.40	1.11 ± 0.39	1.05 ± 0.41	1.11 ± 0.16	1.04 ± 0.18
	D	1.32 ± 0.34	1.30 ± 0.32	1.39 ± 0.37	1.33 ± 0.40	1.46 ± 0.26	1.36 ± 0.27	1.33 ± 0.14	1.27 ± 0.15
5	MB	1.21 ± 0.28	1.15 ± 0.31	1.01 ± 0.42	0.93 ± 0.40	1.04 ± 0.45	0.95 ± 0.45	1.20 ± 0.29	1.13 ± 0.29
	ML	1.17 ± 0.29	1.08 ± 0.31	1.04 ± 0.36	0.98 ± 0.34	1.09 ± 0.42	1.04 ± 0.44	1.18 ± 0.21	1.13 ± 0.19
	D	1.24 ± 0.22	1.22 ± 0.23	1.27 ± 0.40	1.22 ± 0.38	1.32 ± 0.31	1.25 ± 0.33	1.25 ± 0.25	1.13 ± 0.26

No statistical differences were observed in the percentage reduction of dentine between the TradAC and UltraAC groups at any evaluated level in both aspects of the mesial root (*p* < 0.05) (Table 3). However, statistical differences in the percentage reduction of dentine thickness were noted only in the distal root (Table 3), in which Reciproc had a significantly higher percentage reduction of dentine from levels 1 to 4 than XP‐endo Shaper (*p* < 0.05). Nonetheless, no difference was observed between TradAC and UltraAC groups in both aspects of the distal root (*p* > 0.05) (Table 3). In the mesial root, 12 canals (7 TradAC, 5 UltraAC) showed residual dentine thickness below 0.5 mm after preparation. Within TradAC, 4 canals already presented < 0.5 mm thickness before preparation, while in the remaining cases, the initial dentine was relatively thin (0.58–0.65 mm). In the distal root, all instances with < 1 mm thickness had preoperative values below this limit (Figure 1).

**TABLE 3:** Percentage reduction of dentine thickness measured on the mesial and distal aspects of the roots, before and after preparation of the mesiobuccal (MB), mesiolingual (ML) and distal (D) canals with XP‐endo Shaper and Reciproc instruments in mandibular molars prepared with traditional (TradAC) or ultraconservative (UltraAC) access cavities.

Level	Canal	TradAC (*n* = 16)								UltraAC (*n* = 16)							
		XP‐endo Shaper				Reciproc				XP‐endo Shaper				Reciproc			
		Mesial		Distal		Mesial		Distal		Mesial		Distal		Mesial		Distal	
1	MB	3.9	±3.4	15.9	±6.7	6.9	±4.8	19.1	±10.0	6.1	±4.5	16.3	±5.6	6.4	±5.4	21.7	±14.8
	ML	2.1	(3.5)	13.7	(9.2)	4.3	(2.9)	23.6	(14.3)	0.9	(2.1)	7.6	(12.6)	1.2	(4.9)	6.7	(22.4)
	D	**2.5** ^**A**^	**(3.7)**	1.3^A^	(0.3)	**6.5** ^**AB**^	**(14.3)**	0.7^A^	(3.1)	**2.5** ^**A**^	**(7.7)**	1.3^A^	(1.3)	**13.0** ^**B**^	**(2.5)**	0.9^A^	(1.4)
2	MB	4.4	±3.9	15.7	±7.6	7.5	±7.0	18.2	±12.8	5.2	±4.1	15.5	±8.2	5.1	±4.1	24.3	±19.6
	ML	3.4	(2.3)	13.4	±8.1	4.5	(6.8)	24.5	±16.2	1.6	(3.0)	10.2	±10.2	1.2	(7.0)	14.4	±14.1
	D	**1.6** ^**A**^	**(3.3)**	**0.7** ^**A**^	**(1.1)**	**15.8** ^**B**^	**(15.7)**	**0.8** ^**A**^	**(1.1)**	**1.0** ^**A**^	**(3.8)**	**2.0** ^**B**^	**(1.1)**	**14.3** ^**B**^	**(2.8)**	**0.8** ^**A**^	**(0.5)**
3	MB	5.6	±3.3	10.6	±6.4	9.0	±6.3	14.3	±10.7	6.5	±4.7	14.0	±9.3	6.7	±5.4	13.2	±11.6
	ML	5.2	(3.0)	9.3	(16.8)	7.4	(7.5)	15.0	(14.2)	1.8	(3.8)	3.9	(14.1)	4.5	(9.5)	15.9	(18.5)
	D	**1.3** ^**A**^	**(1.8)**	1.0^A^	(0.5)	**12.7** ^**B**^	**(9.2)**	1.7^A^	(3.9)	**1.2** ^**A**^	**(1.7)**	3.6^A^	(5.4)	**14.1** ^**B**^	**(4.7)**	1.4^A^	(2.2)
4	MB	6.7	±2.8	3.3	(5.8)	11.2	±6.1	13.7	(11.5)	9.3	±5.1	5.3	(11.4)	9.1	±9.0	5.1	(10.4)
	ML	7.9	±4.9	2.3	(7.0)	10.4	±5.2	7.3	(9.1)	4.8	±4.8	5.3	(10.8)	8.1	±5.3	1.7	(12.3)
	D	**1.6** ^**A**^	**(1.9)**	0.8^A^	(0.4)	**7.7** ^**B**^	**(5.9)**	3.7^A^	(6.9)	**1.3** ^**A**^	**(1.9)**	4.2^A^	(10.2)	**10.3** ^**B**^	**(6.3)**	3.7^A^	(7.2)
5	MB	5.9	±4.3	5.8	±4.2	9.0	±6.1	8.0	±7.3	9.8	±5.6	10.8	±8.9	10.1	±9.7	6.3	±6.9
	ML	7.9	±6.5	10.8	(6.8)	9.5	±5.1	4.9	(7.4)	5.1	±4.5	2.3	(11.5)	7.7	±4.6	2.3	(5.8)
	D	1.1^A^	(1.8)	**0.8** ^**A**^	**±1.5**	6.5^A^	(10.3)	**2.8** ^**AB**^	**(5.1)**	1.9	(3.9)	**4.8** ^**B**^	**(4.9)**	7.0^A^	(7.8)	**7.8** ^**B**^	**(8.9)**

*Note:* Different uppercase letters signify statistically significant differences among groups for the same aspect of the root concerning the percentage reduction of dentine thickness after preparation at each root level (*p* < 0.05). Bold letters are used to enhance readability and highlight rows with statistical differences. Values are presented as mean ± standard deviation for parametric data or as median (interquartile range) for nonparametric data.

**FIGURE 3:**
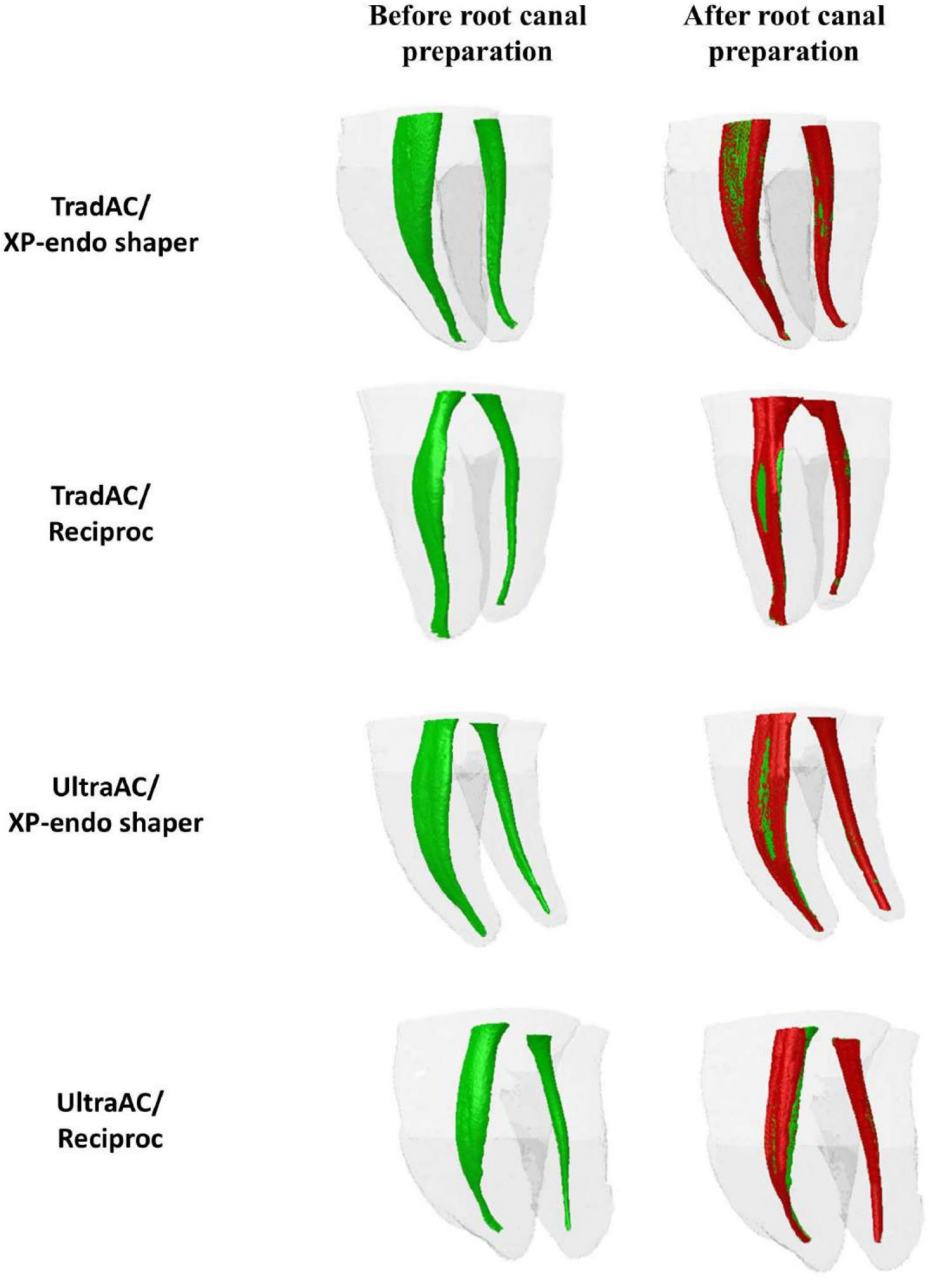
Representative 3D micro‐CT images of mandibular molars prepared with TradAC and UltraAC, showing the root canals before (in green) and after (in red) preparation using the Reciproc and XP‐endo Shaper systems.

Regarding the shaping ability, no significant differences were observed between Reciproc and XP‐endo Shaper in terms of changes in surface area, canal volume, or the percentage of untouched canal walls, in teeth prepared with either TradAC or UltraAC (*p* > 0.05) (Table 4; Figure 3), and no instrument fractures occurred in any specimen after preparation.

**TABLE 4:** Mean (±standard deviation) or median (interquartile range) surface area (in mm^2^), volume (in mm^3^), and untouched canal walls (in %) after preparation of the mesial and distal canals of mandibular molars using XP‐endo Shaper and Reciproc instruments in teeth prepared with a traditional access cavity (TradAC) or an ultraconservative access cavity (UltraAC).

		Parameters	TradAC (*n* = 16)				UltraAC (*n* = 16)			
			XP‐endo Shaper		Reciproc		XP‐endo Shaper		Reciproc	
			Mesial	Distal	Mesial	Distal	Mesial	Distal	Mesial	Distal
Apical third	Preoperative	Surface area	13.4 ± 6.5	8.1 (2.5)	11.3 ± 4.5	5.3 (2.0)	12.4 ± 3.4	5.3 (2.0)	15.8 ± 3.8	6.9 (1.8)
		Volume	1.2 ± 0.8	0.8 (0.4)	0.9 ± 0.4	0.4 (0.3)	0.9 ± 0.3	0.4 (0.3)	1.3 ± 0.4	0.7 (0.3)
	Postoperative	Surface area	14.6 ± 6.3	8.6 (2.2)	13.9 ± 4.2	6.6 (1.5)	13.5 ± 3.2	6.6 (1.5)	17.4 ± 3.4	7.9 (2.6)
		Volume	1.5 ± 0.8	1.0 ± 0.6	1.3 ± 0.5	0.7 ± 0.3	1.2 ± 0.3	1.2 ± 0.7	1.6 ± 0.4	1.0 ± 0.3
		Untouched walls	24.4 (14.2)	15.5 (9.1)	28.8 (13.7)	14.2 (4.5)	28.8 (13.7)	14.2 (4.5)	36.3 (5.7)	18.1 (14.0)
Root canal	Preoperative	Surface area	52.1 ± 14.4	32.4 ± 8.5	45.6 ± 9.9	28.6 ± 10.0	47.7 ± 5.6	37.1 ± 9.7	54.6 ± 18.5	36.1 ± 10.5
		Volume	5.7 ± 2.2	5.0 (2.0)	4.9 ± 2.3	3.7 (4.7)	4.9 ± 0.9	7.0 (2.1)	6.1 ± 2.5	7.0 (3.9)
	Postoperative	Surface area	55.1 ± 13.9	33.6 ± 8.6	54.2 ± 10.8	32.2 ± 8.8	54.1 ± 6.0	38.4 ± 9.4	61.4 ± 15.5	38.9 ± 10.4
		Volume	7.3 ± 2.1	5.4 (1.8)	7.6 ± 2.8	4.5 (3.6)	6.1 ± 1.0	7.9 (2.5)	8.3 ± 2.5	8.4 (4.1)
		Untouched walls	18.1 (14.3)	12.5 ± 6.8	22.8 (11.4)	10.8 ± 7.7	27.1 (26.8)	14.6 ± 12.5	30.4 (17.7)	14.6 ± 6.5

*Note:* Shaping ability parameters were assessed in the apical third (from the apical foramen up to 4 mm in the coronal direction) and in the root canal extending from the foramen to 10 mm coronally. No significant differences were observed when comparing the same root canals prepared with either Reciproc or XP‐endo Shaper (*p* > 0.05).

## 4 Discussion

Shaping procedures in endodontics progress as new NiTi instruments are consistently introduced to the market. In recent years, there has been a significant shift in root canal preparation protocols towards embracing the principles of minimally invasive endodontics, emphasising the preservation of dentine in both the crown and pericervical region of teeth. In line with this trend, numerous clinicians advocate for a reduction in access preparation size, promoting what is known as the conservative access cavity. Concurrently, manufacturers are actively developing instruments with smaller dimensions, such as the XP‐endo Shaper, aligning with the goal of minimising dentine removal. Therefore, the present study aimed to evaluate how the different access cavity types (TradAC vs. UltraAC), alongside instrumentation protocols with Reciproc and XP‐endo Shaper instruments, impact the percentage reduction of dentine thickness in the pericervical region of mandibular molars. The results showed that access cavity design and instrument type did not influence dentine reduction or shaping ability, leading to acceptance of the null hypothesis. This finding suggests that the access cavity design may not be the primary determinant of dentine preservation, at least within the parameters of this in vitro study. This contradicts the narrative that has driven the trend towards minimally invasive endodontics [3, 5, 6] and aligns with recent findings that have questioned the purported benefits of contracted access cavities [8, 9, 10].

In mandibular molars, the distal surface of the mesial root is particularly susceptible to perforation due to thin dentine, a region historically termed the “danger zone” [17, 24]. Its anatomy and response to canal preparation have been studied using both destructive [25, 26] and non‐invasive approaches [16, 25, 27, 28, 29], with minimum dentine thickness reported between 0.67 mm [16] and 1.5 mm [28]. In this study, preoperative dentine thickness at the pericervical area ranged widely (Figure 1), highlighting the importance of sample selection and baseline comparability [30]. Therefore, efforts were undertaken to ensure that the groups were comparable in this aspect by establishing a uniform and dependable baseline. This was achieved by acquiring quantitative data through prior scanning of all samples, a process that enhanced the internal validity of the method and minimised the anatomical biases typically associated with study outcomes [30]. As a consequence, no significant difference was noted in the percentage reduction of dentine thickness at the pericervical area of mesial roots when comparing groups with different access cavity designs. Additionally, despite employing two preparation protocols utilising instruments with distinct geometric characteristics and kinematics (XP‐endo Shaper and Reciproc), no difference was also observed between them in this aspect.

The XP‐endo Shaper is made from a proprietary alloy known as MaxWire (FKG Dentaire SA) which incorporates a unique Martensite–Austenite electropolish‐fleX technology, allowing it to exhibit differential responses at various temperatures. The instrument has an initial taper of 0.01 in its Martensite phase when cooled. However, upon exposure to body temperature (35°C), its alloy transitions to its Austenite phase, wherein the taper expands to 0.04 due to molecular memory [31, 32, 33, 34]. According to the manufacturer's specifications, when operated at 800 rpm, the adaptive core design of the file (ISO size 30/0.01) enables it to initiate shaping at ISO size 15 and progress to achieve ISO size 30. Additionally, it can increase the taper from 0.01 to at least 0.04, resulting in a final canal preparation size of a minimum 30/0.04 size [35], though this may vary depending on canal anatomy [36].

One of the objectives of this study was to compare the shaping ability of the XP‐endo Shaper, an instrument specifically designed to “preserve the canal structure to a remarkable extent” [34], with a conventional instrument, the Reciproc R25, in mandibular molars prepared with TradAC or UltraAC. At first glance, this comparison appears unbalanced due to the difference in instrument dimensions, particularly in the preparation of the distal canal, where a Reciproc R40 was used as the master apical file. However, previous research has demonstrated that the XP‐endo Shaper is capable of enlarging canals beyond its minimum specification of 30/0.04. In a study evaluating long oval‐shaped mandibular incisors, the mean major diameter at the working length increased from 0.36 to 0.45 mm after preparation with the XP‐endo Shaper [36]. Moreover, another micro‐CT study in maxillary molars demonstrated that the XP‐endo Shaper produced sufficient enlargement to accommodate 0.04‐tapered gutta‐percha cones up to size 50, particularly in broader canals [37]. This phenomenon can be explained by the inherent anatomical differences among root canals. Naturally wider canals, such as the distal canal of mandibular molars, provide more space for the instrument, allowing for greater expansion compared to narrower canals, such as the mesial canal. As a result, the instrument can exceed the minimum size specified by the manufacturer. The present results are consistent with these findings, as no differences were observed in any of the shaping ability parameters tested for either system in both mesial and distal roots (Table 4; Figure 3). Furthermore, the similarities in the shaping ability outcomes of both systems can be attributed to their comparable effectiveness in maintaining centralization within the root canal. In a study investigating canal transportation at the apical third caused by XP‐endo Shaper and Reciproc R25 instruments and comparing their effects on the displacement of the gravity centre of the root canal axis using micro‐CT, no significant differences were found in their mean transportation for both mesial canals [38].

Preoperative dentine thickness in the distal root ranged from 0.64 to 2.35 mm, roughly double the minimum thickness of the mesial root, though maximum values were similar between roots (Tables 1 and 2). This highlights the greater vulnerability of the mesial root to excessive dentine removal. Despite these variations, the shaping procedures preserved dentine thickness within a similar range in both roots, reinforcing the importance of instrumentation strategies that minimise unnecessary dentine loss, particularly in weaker regions. Canals prepared with Reciproc demonstrated significantly higher reductions in different levels of the root compared to XP‐endo Shaper. This disparity can be readily attributed to the larger size of the Reciproc instrument used in the distal root (R40) compared to that in the mesial root (R25). Additionally, it is important to emphasise that in the XP‐endo Shaper group, the single file was used during root canal preparation to test the manufacturer's recommendation, without the insertion of other instruments to avoid introducing another element of bias. Nevertheless, once more, it is worth noting that the type of access cavity (TradAC vs. UltraAC) did not influence the outcomes.

Some studies have proposed threshold values for dentine thickness to safeguard the mechanical integrity of roots, with some authors suggesting values below 0.3 mm [39], while others advocate for a threshold of 0.5 mm [40]. Although the arbitrary nature of these values, 0.5 mm was adopted as a threshold in this study to enable comparison with existing literature. Despite the lack of a statistically significant difference in overall dentine preservation between the access cavity designs, it is important to note that in twelve of the mesial canals, the final dentine thickness was less than 0.5 mm. This is a critical threshold, as it has been associated with an increased risk of root fracture [20, 24]. In four of these cases, the initial dentine thickness was already below this threshold, highlighting the importance of pre‐operative assessment of root anatomy. In the remaining eight cases, the initial dentine thickness was only slightly above the 0.5 mm threshold (0.58–0.65 mm), suggesting that even with conservative instrumentation, the risk of excessive thinning remains. A similar occurrence was noted by other authors in mandibular molars that were prepared with TradAC, where the mesial root canals were enlarged using other instruments such as ProTaper Next X2 and X3 [41] and Hero 642 sizes 30/0.04 and 30/0.06 [42]. In line with the current findings, these studies reported pre‐operative mean dentine thickness greater than 0.88 mm, reinforcing that the initial thickness measured in mesial roots of mandibular molars holds greater significance in predicting reduction below the critical threshold than the type of access preparation or preparation protocol. It is important to consider that teeth with thin preoperative dentine thickness were not replaced during sample selection because the statistical analyses in this study focused on comparing the percentage reduction in dentine thickness, rather than the absolute values of remaining dentine thickness, making preoperative values irrelevant for the comparisons. This approach is particularly important because balancing groups based on preoperative dentine thickness presents significant challenges and is nearly unfeasible, given the natural variability in dentine thickness among individual teeth and the difficulty in standardising this factor across groups. So, the present findings suggest that clinicians can enhance their decision‐making process to choose the most suitable instruments to enlarge root canals of mandibular molars by considering the dimensions of preoperative dentine. However, further research is still necessary to determine precise critical dentine thickness values that could compromise the integrity of root structure.

The main limitation of this study is the lack of instruments with varying taper sizes and rotary kinematics, as well as the evaluation of other tooth groups such as maxillary molars. On the other hand, the primary strengths of this study included performing measurements using a gold standard 3D approach (micro‐CT), which ensured comparability between groups in terms of dentine thickness before the experimental procedures. The use of mandibular molars with a mesial canal system exhibiting a Type V isthmus [22] also provides valuable insights into how the Reciproc and XP‐endo Shaper instruments perform in a less commonly studied anatomical configuration, as most previous studies evaluating root canal preparation in molars with varying access cavity designs have predominantly focused on teeth with mesial canal configurations classified as Types II and IV [5]. Furthermore, mounting the extracted teeth on a dental manikin and performing the experimental procedures under ergonomic working positions simulated the clinical setting more realistically. Further research is required to achieve a more comprehensive understanding of how access cavity design may impact dentine thickness in different tooth types, particularly those in which root canals are prepared using instruments with diverse sizes, designs, and kinematics.

## 5 Conclusion

Within the limitations of this laboratory study, access cavity design had no influence on dentine preservation, while the choice of instrument affected dentine reduction only in the distal root.

## Author Contributions

Renata Muniz Alvez Cruz: execution of the experimental design; data analysis; writing. Ana Flávia Almeida Barbosa: planning and execution of the experimental design; data analysis; writing. Carolina Oliveira de Lima: planning and execution of the experimental design; data analysis; writing. Ricardo Tadeu Lopes: data analysis. Marco Aurélio Versiani: idea, hypothesis; planning and execution of the experimental design; data analysis; writing, managing and proofreading the manuscript. Emmanuel João Nogueira Leal da Silva: idea, hypothesis; planning and execution of the experimental design; data analysis; writing, managing and proofreading the manuscript. Luciana Moura Sassone: idea, hypothesis; planning and execution of the experimental design; data analysis; writing, managing and proofreading the manuscript. All authors have contributed significantly to this study and the preparation of the manuscript. Furthermore, all authors have reviewed and approved the final version of the manuscript, agreeing to its content and submission.

## Funding

This work was supported by Conselho Nacional de Desenvolvimento Científico e Tecnológico; Coordenação de Aperfeiçoamento de Pessoal de Nível Superior.

## Ethics Statement

This study was approved by the Research Ethics Committee (protocol 48541321.8.0000.5259).

## Conflicts of Interest

The authors declare no conflicts of interest.

## Supporting information


**Figure S1:** PRILE flowchart.

## Data Availability

The data that support the findings of this study are available from the corresponding author upon reasonable request.
